# Lithotomy on a Female

**Published:** 1851-03

**Authors:** Frank H. Hamilton

**Affiliations:** Prof. of Surgery in the Medical Department of the University of Buffalo


### BUFFALO MEDICAL JOURNAL

AND

### MONTHLY REVIEW.

**VOL. 6.** MARCH, 1851. **NO. 10.**

### ORIGINAL COMMUNICATIONS.

ART. I.—*Lithotomy on a Female.* Reported by FRANK H. HAMILTON, M. D., Prof. of Surgery in the Medical Department of the University of Buffalo.

Caroline Friesz, of Buffalo, aged 23 years, has had symptoms of stone in the bladder since she was three years old. During the last few months she had suffered greatly from incontinence, and pain, and her health had become so much impaired that she was confined to her bed. Several days previous to the operation, she had been unable to sleep, and it was evident that without relief she must soon die.

On the 19th of March, I made an operation for the removal of the stone. Having placed the patient for a subpubic operation, and having committed the hands and feet to assistants, for she was not tied, I introduced a narrow, probe-pointed, straight bistoury, without a director, into the bladder, and cut outward and downward.

I adopted a precaution in this stage of the operation, similar to what I have before recommended and practised in making the same operation upon the male. In the male, I recommend keeping the forefinger of the left hand in the rectum, while the thumb of the same hand follow the wound, to avoid cutting the rectum. The danger of cutting the vagina in the female is quite as great, and I therefore kept the forefinger of the left hand in the vagina, and the thumb of the same hand in the wound. Still more effectually to avoid opening the vagina, the edge of the bistoury was at first directed *outward* and then downward. If the directions given to cut "outward and downward" are literally followed, especially in females who have borne children, where the urethra fairly sinks into the vagina, a wound of the latter will often be inevitable.

The stone was easily seized, but could not be disturbed from its bed at the fundus of the bladder. It was then broken, and after very protracted efforts, about two-thirds of the whole stone was removed. During a portion of this time I was assisted by Dr. Alden Sprague of this city, and by the counsel of other medical gentlemen who were present. Although the stone was large, it was quite evident that it was not for want of a sufficient opening that we failed to remove it, but because it was encysted. We at length desisted, leaving a portion of the stone yet within the bladder. During the whole time occupied in the operation, the patient was kept more or less completely under the influence of chloroform; one ounce was consumed! Before the operation was completed, she gave signs of sinking, and we commenced the free administration of brandy. She soon rallied, and remained quiet and comfortable for some hours, when a peritoneal inflammation supervening, she died on the 3d day after the operation.

*Autopsy.*—Serum and lymph in the cavity of the peritoneum. The portion of stone which had not been removed found situated in the fundus of the bladder, and could not be dislodged by pressure upon it, or by seizing it with the forceps, the body of the bladder being contracted to the extent of two or three inches from the neck upward, so as to resemble the neck itself. This narrow portion was knobby and irregular upon its inner surface. The fundus, containing the unremoved stone, was more smooth, but had several indentations of three or four lines in depth, into which corresponding prolongations from the calculus were fixed.

To have removed this calculus entire it would have been necessary to have made an incision along the narrowed and thickened body of the bladder until the disturbed fundus was reached and laid open, a proceeding which must have rendered a fatal urinary infiltration inevitable.

I was also able to determine in the autopsy, that the fundus of the bladder lay too high to have been reached from the vagina. The entire stone, including the fragments which were removed in the operation, weighed four ounces.

Its composition is ammonio-phosphate of magnesia and lime with traces of lithates.

